# Association of Kinase-Insert-Domain-Containing Receptor Polymorphisms with Glioma Susceptibility in a Chinese Population: A Hospital-Based Case-Control Study

**DOI:** 10.1155/2023/8808422

**Published:** 2023-04-18

**Authors:** Zhi-Fa Huang, Wei Zhu, Chen Wang, Li-Dong Mo, Hui-Ling Huang, Xiao-Guang Tong

**Affiliations:** ^1^Clinical College of Neurology, Neurosurgery and Neurorehabilitation, Tianjin Medical University, Tianjin, China; ^2^Department of Neurosurgery, Tianjin Huanhu Hospital, Tianjin, China; ^3^Tianjin Key Laboratory of Cerebral Vascular and Neurodegenerative Diseases, Tianjin Neurosurgical Institute, Tianjin Huanhu Hospital, Tianjin, China

## Abstract

**Background:**

Gliomas are the most common malignant tumors of the central nervous system. However, the inherited genetic variation in gliomas is presently unclear. Therefore, this study investigated the association of the rs2071559 and rs2239702 gene polymorphisms with glioma susceptibility in Chinese patients.

**Methods:**

In this study, a case-control approach was used to compare and analyze whether two genes, rs2071559 and rs2239702, were associated with the risk of glioma formation.

**Results:**

The cases and controls were matched for sex, smoking status, and family history of cancer using single nucleotide polymorphisms. Specific rs2071559 and rs2239702 alleles were found much more frequently in the glioma group than in the control group (*P* < 0.001 and *P* = 0.014, respectively).

**Conclusions:**

These findings suggest that specific rs2071559 and rs2239702 polymorphisms are associated with a higher risk of glioma development; the risk allele is C in rs2071559 or A in rs2239702. Moreover, the kinase-insert-domain-containing receptor may act as a suppressor of tumor progression.

## 1. Introduction

Cancer is a major global health issue, and its worldwide incidence and mortality continue to increase rapidly; in China, cancer is the leading cause of death [1, 2]. China is the most populous country in the world, with an estimated population of 1.42 billion; in 2020, there were 4.5 million cancer patients and over 3 million cancer-related deaths. In addition, cancer accounts for over 67 million disability-adjusted life years in China [3]. Although primary brain tumors account for only an estimated 1.8% of malignancies, worldwide, they account for a disproportionate burden of cancer mortalities because of their high fatality rate [4].

Gliomas (including astrocytomas, oligodendrogliomas, ependymomas, and a variety of rare histologies [5]) are the most common malignant tumors of the central nervous system, accounting for up to 80% of all malignant brain tumors. According to the World Health Organization (WHO) classification, gliomas are graded from 1 (slow-growing tumors) to 4 (fast-growing tumors) [6, 7]. These tumors can have profound effects on physical, neurocognitive, and social functioning, beginning at an early stage in patients with high-grade, fast-growing tumors [8]. The neurocognitive effects of the disease, accompanied by the increased dependency and social isolation, can result in an enormous burden on patient relationships with family members/care providers [9]. The main reason for this tragic situation is a lack of understanding of the etiology of this disease. To date, exposure to ionizing radiation is the only exogenous factor that has been established as contributing to glioma formation [10]. However, the role of inherited genetic variation in gliomas is presently unclear.

Gliomas are rich in blood vessels, and angiogenesis is a prerequisite for tumor growth [11]. Vascular endothelial growth factor (VEGF) and VEGF receptor (VEGFR) are thought to play major roles in tumor angiogenesis [11]. VEGFR is a typical transmembrane integral protein divided into VEGFR-1 (Flk-1), VEGFR-2 (Flt-1/kinase-insert-domain-containing receptor (KDR)), and VEGFR-4 (Flt-3) [12]. VEGFR-2 is generally known to play a principal role in mediating VEGF-induced responses [13, 14]. Importantly, VEGFR-2 is the most important receptor for angiogenesis during tumor invasion [15]. KDR overexpression has been studied in relation to several different types of cancer, including lung [16], colon [17], uterine and ovarian [18], and breast [19] cancers. Moreover, there is a significant correlation between KDR expression and vasculogenesis and angiogenesis in gliomas [20]. However, little is known regarding the association between KDR single nucleotide polymorphisms (SNPs) and glioma susceptibility. Both the rs2071559 and rs2239702 polymorphisms are located in the promoter region of KDR, and certain studies have found that this polymorphism affects mRNA and protein expression [21].

Thus, we aimed to determine if there was an association between the rs2071559 and rs2239702 polymorphisms and susceptibility to glioma development using a case-control study in a Chinese population.

## 2. Methods

### 2.1. Study Population

Patients with pathologically confirmed gliomas and of Han Chinese origin were consecutively recruited from October 2009 to February 2011 in the Department of Neurosurgery at the Huashan Hospital of Fudan University (Shanghai, China). Although there were no restrictions on age, sex, or histology, the exclusion criteria included previous chemotherapy and radiotherapy for unknown disease conditions and a self-reported history of cancer. The controls, trauma patients from the Tianjin Huanhu Hospital and Huashan Hospital, had no self-reported history of cancer, central nervous system-related disease, or history of radiotherapy/chemotherapy.

To obtain detailed information on demographic factors, smoking status, family history of cancer (fhc), and health characteristics, each consenting patient was interviewed using a structured questionnaire. The epidemiological questionnaire was designed with reference to the Brain Tumor Epidemic Questionnaire (MD Anderson Cancer Center; Houston, TX, USA). After strict training, the investigators conducted face-to-face inquiries and investigations on the basis of the informed consent of the respondents and accurately recorded them. The contents of the epidemiological investigation included general demographic characteristics (age, sex, and place of origin), occupation, history of major diseases, family history of tumors in first-degree relatives, smoking status, and dietary nutritional status, and clinical data (including diagnoses and treatments). Participants were classified as nonsmokers, former smokers, and current smokers according to their smoking status. In this study, smokers were defined as those who smoked at least one cigarette per day for more than one year. At the time of the survey, those who had quit smoking for more than one year were defined as former smokers. The glioma types were roughly divided into three types according to their pathological origin: glioblastoma, astrocytoma other than glioblastoma (mainly diffuse and anaplastic astrocytoma and diffuse and anaplastic astrocytomas), and other types of gliomas (including oligodendroglioma, anaplastic oligodendroglioma, ependymoma, medulloblastoma, choroid plexus papilloma, and mixed glioma) [22]. All questionnaire contents and responses were digitally archived. After checking, error correction, and conversion assignment, an information database for glioma cases and normal controls was established.

This study was approved by the Fudan University Ethics Committee for Human Subject Research and each participant provided written informed consent. For minors (individuals less than 18 years of age), signed informed consent was obtained from a guardian/parent.

### 2.2. Blood Sample Collection

A 5 mL peripheral venous blood sample was collected from each patient and placed into a tube containing a citrate-dextrose anticoagulant. All samples were maintained at room temperature prior to analysis.

### 2.3. Gene Variant Selection and Genotyping

Searching for functional variants in the promoter regions of a gene is important because the promoter region functions in regulating gene transcription and production. Previous studies demonstrated that VEGFR-2 promoter polymorphisms may alter susceptibility to coronary heart disease, stroke, and atopy [21, 23, 24]. rs2071559 and rs2239702 are two of the 16 SNPs in the KDR. They are located to each other in the promoter region of KDR gene. rs2071559 is located in the binding site of the promoter region of the KDR of ribonucleoprotein (a putative transcriptional factor). This SNP (−604T > C) is predicted to lead to a lower binding efficiency for the promoter region of KDR and its corresponding transcription factor, downregulating KDR expression, and decreasing the levels of KDR. The rs2071559 polymorphism is associated with lymphatic metastasis in patients with nasopharyngeal carcinoma (NPC) and in those with reduced susceptibility to atherothrombotic stroke [23, 25]. Therefore, these two variants of the promoter region were included in the present study.

Venous blood (2 mL) from each patient was collected in tubes containing ethylenediaminetetraacetic acid. White blood cell fractions were processed for genomic DNA extraction using the Qiagen Blood Kit (Qiagen, Chatsworth, California, USA). Then, the genomic DNA was diluted to a concentration of 15–20 ng/*μ*L for genotyping assays. Polymerase chain reaction was used to amplify polymorphism spanning fragments, and both variants (rs2071559 and rs2239702) were genotyped using the Mass ARRAY iPLEX platform (Sequenom, San Diego, CA, USA) using an allele-specific matrix-assisted laser desorption/ionization time-of-flight mass spectrometry assay; the assays were conducted without the analyst having knowledge of the control or case status of the sample. MassARRAY Assay Design software, version 3.1 (Sequenom) was used to design primers for the amplification and extension reactions, and SNP genotypes were obtained according to the iPLEX protocol provided by the manufacturer. Genotyping quality was examined using a detailed quality control procedure consisting of a >95% successful call rate with duplicate calling of genotypes, internal positive control samples, and subsequent Hardy–Weinberg equilibrium testing.

### 2.4. Statistical Analysis

Deviation from the Hardy–Weinberg equilibrium was assessed using Fisher's exact test for each SNP among the controls, and *χ*^2^ tests were used to compare the differences in demographic characteristics as well as frequency distributions of alleles and genotypes between the controls and cases. The multivariate logistic regression analyses were conducted to estimate the odds ratios (ORs) and 95% confidence intervals (CIs) of SNPs, adjusted for sex and age; sex, age, and family history of cancer; and sex, age, smoking status, and family history of cancer. The reference group had the most common genotype among the controls. All statistical tests were 2-sided. For the two SNPs in the KDR, we used Haploview (Broad Institute; Cambridge, MA, USA) to estimate pairwise linkage disequilibrium in the control subjects. We used the software package HaploStats (https://www.mayo.edu/hsr/Sfunc.html) to perform the haplotype analysis. Patients with glioma were stratified into three subgroups according to the lesion histology: glioblastoma, other than the glioblastoma astrocytoma (diffuse and anaplastic astrocytomas), and other types of gliomas (including oligodendroglioma, anaplastic oligodendroglioma, ependymoma, medulloblastoma, choroid plexus papilloma, and mixed glioma). Subgroup analyses were performed to estimate the specific ORs based on histology. For the risk alleles that were independently associated with increased glioma risk, their cumulative effect was assessed by counting the number of risk alleles per person from the two SNPs of the KDR (categories were 0–1, 2, 3, and 4). SPSS software (Version 17.0; SPSS; Chicago, IL, USA) was used to perform all statistical analyses unless otherwise indicated.

## 3. Results

### 3.1. Baseline Characteristics

In total, 465 glioma patients and 527 cancer-free control patients were enrolled in the study. The characteristics of the included patients are summarized in Table 1. Overall, the cases and controls appeared to be adequately matched for sex, smoking status, and fhc (*P* = 0.327, 0.572, and 0.378, respectively). Although there was no evidence of general demographic difference between the patients with glioma and the control patients, there was a statistically significant difference in age between the groups (*P* = 0.046). The mean (± standard deviation) age of the patients with gliomas was 42.22 ± 15.46 years and 40.20 ± 16.30 years in the control patients; males accounted for 58.5% of the patients with gliomas and 55.4% of the control patients.

Among the 465 patients with gliomas, the tumors were classified as astrocytic gliomas (173, 37.2%), glioblastomas (159, 34.2%), ependymomas (67, 14.4%), oligodendrogliomas (47, 10.1%), and mixed gliomas (19, 4.1%). Moreover, 210 patients demonstrated low-grade, slow-growing tumors, and 255 demonstrated high-grade, fast-growing tumors, according to the WHO classification (Table 2).

### 3.2. Hardy–Weinberg Equilibrium

The observed allele frequencies are presented in Table 3 using SNP information. In the control population, both polymorphisms demonstrated Hardy–Weinberg equilibrium (*P* > 0.05) (Table 3).

### 3.3. SNP Genotyping Results

The minor allele frequencies among the controls were in the range of the published allele frequencies for the Han Chinese population (Table 3). The cases and controls were matched for sex, smoking status, and fhc for each SNP. There were differences in the distribution of the rs2071559 and rs2239702 alleles (*P* < 0.001 and 0.014, respectively) between the cases and controls.

The genotypic distributions of rs2071559 and rs2239702 in the case and control patients are summarized in Table 4. The C allele of rs2071559 was present in 38.6% of cases and in 32.2% of the controls, whereas the A allele of rs2239702 was present in 18.9% of cases and in 14.8% of the controls. The frequencies of the rs2071559 T/T, C/T, and C/C genotypes were 38.7%, 45.4%, and 15.9% in controls and 49.5%, 40.6%, and 9.9% in the cases, respectively. The frequencies of the rs2239702 G/G, G/A, and A/A genotypes were 67.7%, 26.7%, and 5.6% in the controls and 72.9%, 24.6%, and 2.5% in the cases, respectively (Table 4).

### 3.4. Association between Individual SNP and Glioma Risk in the Univariable Analysis

Overall, Table 4 shows that the C allele of rs2071559 was associated with an increased risk of glioma compared with the T allele (OR = 1.46; 95% CI, 1.21–1.75; *P*  < 0.001), and the A allele of rs2239702 was associated with an increased risk of glioma compared with the G allele (OR = 1.34; 95% CI, 1.06–1.70; *P* = 0.014). For rs2071559, the C/T and C/C genotypes were both associated with an increased risk of glioma development (OR = 1.43; 95% CI, 1.09–1.87 and OR = 2.06; 95% CI, 1.38–3.09, respectively), using the T/T genotype as a reference. For rs2239702, the A/A genotype was associated with an increased risk of glioma development (OR = 2.44; 95% CI, 1.23–4.82), using the G/G genotype as a reference. For rs2071559, the dominant genetic model showed that T/T was significantly associated with glioma risk in the univariate analysis (OR = 1.55, 95% CI, 1.21–2.00, *P* = 0.001). A recessive genetic model also showed that C/C was associated with glioma risk in the univariate analysis (OR = 1.73, 95% CI, 1.18–2.53, *P* = 0.004). For rs2239702, a recessive genetic model showed that A/A was associated with glioma risk in the univariate analysis (OR = 2.34; 95% CI, 1.19–4.61, *P* = 0.012).

### 3.5. Association between Individual SNP and Glioma Risk in the Multivariate Analysis

For rs2071559, the C/T and C/C genotypes were both associated with an increased risk of glioma formation (adjusted OR = 1.45; 95% CI, 1.10–1.92 and adjusted OR = 2.04; 95% CI, 1.35–3.09, respectively), using the T/T genotype as a reference. For rs2239702, the A/A genotype was associated with an increased risk of glioma development (adjusted OR = 2.50; 95% CI, 1.25–5.01), using the G/G genotype as a reference. For rs2071559, the dominant genetic model showed that T/T was significantly associated with glioma risk in the multivariate model (adjusted OR = 1.57; 95% CI, 1.20–2.04, *P* = 0.001). A recessive genetic model showed that C/C was associated with glioma risk in the multivariate model (adjusted OR = 1.69; 95% CI, 1.15–2.50; *P* = 0.008). For rs2239702, a recessive genetic model showed that A/A was associated with glioma risk in the multivariate model (adjusted OR = 2.63; 95% CI, 1.34–5.35; *P* = 0.011) (Table 5).

## 4. Discussion

In this study, we assessed the contribution of two potentially functional variants of the KDR gene to the risk of glioma in the Han Chinese population. We found that both variants in the promoter region were associated with glioma risk. In the present study, a haplotype analysis revealed that the haplotype containing the risk allele (C in rs2071559 or A in rs2239702) is associated with increased glioma risk. In addition, we showed that the CC and CT genotypes of rs2071559 and the AA homozygote of rs2239702 showed a significantly increased association with the risk of glioma. The positive association of variants rs2071559 and rs2239702 with glioma risk remained significant after adjusting for both sex and age or adjusting for sex, age, smoking status, and fhc. These findings suggest that genetic variants of VEGFR-2 may be associated with glioma development in the Chinese population.

The study of inherited susceptibility to gliomas has been one of the most important areas of research during the past decade. We can better understand the biological mechanism of glioma development and identify potential targets for therapeutic interventions if susceptibility genes can be identified. The development of new capillary networks is necessary for glioma growth and tumor angiogenesis is thought to be mediated by soluble factors released from tumor cells. These factors act on the endothelial cells in a paracrine manner. VEGF is a prime regulator of tumor angiogenesis and vasculogenesis, and KDR is a receptor for various VEGF isoforms. Previous studies have shown that VEGF and the high-affinity VEGF receptor KDR are key regulators of tumor angiogenesis. Strategies to block VEGF/KDR signaling have been successfully used to inhibit experimental tumor growth and indicate that KDR is the prime signaling VEGF receptor involved in the proliferating tumor endothelium [26].

Some reports on the relationship between KDR and glioma susceptibility have been published. Coexpression of VEGF and KDR commonly occurs in astrocytoma and glioblastoma cells [27]. KDR is upregulated in the tumor vasculature of anaplastic oligodendrogliomas, glioblastoma multiforme, and ependymomas with necrosis, whereas oligodendroglioma, grade II astrocytomas, and anaplastic astrocytomas tend to express weak-to-nondetectable signals [28]. Moreover, significantly elevated levels of KDR mRNA have been reported in malignant tumor endothelia [29]. These previous studies suggest that overexpression of KDR may influence cell activity in brain tissues and consequently contribute to glioma formation.

Although there are few reports on the relationship between KDR SNPs and glioma susceptibility, some studies have investigated the predisposing role of rs2071559 in other diseases, with conflicting results. Millauer et al. demonstrated that KDR is generally involved in the growth of many solid tumors, such as mammary, ovarian, and lung carcinomas, as well as glioblastomas [30]. The T/T genotype of rs2071559 was reported to be associated with an increased risk of age-related macular degeneration and coronary heart disease [21, 31]. Compared with the homozygous wild-type genotype, variant-containing genotypes exhibited a borderline increased relapse rate in patients with colorectal cancer [32]. For rs2071559, Sjostrom et al. found an association between the major allele and survival time, with shorter survival in heterozygote patients compared with homozygote patients [33]. Chen et al. found that the C/C homozygote of rs2071559 is associated with an increased risk of the glioma development [34].

Therefore, we studied the association of the rs2071559 and rs2239702 polymorphisms with glioma susceptibility in a relatively large sample size from a hospital population of people sharing a common ethnicity (465 cases and 527 controls). However, our study has several limitations, including selection bias, effects of multiple environmental factors on genes, and the representativeness of the studied SNPs in VEGFR-2. Although the controls and cases were matched by sex, smoking status, and fhc to limit potential selection bias, other selection biases cannot be ruled out. At present, owing to the lack of data on many environmental carcinogenic factors in the sample, we cannot clarify the relationship between environmental exposure and glioma susceptibility. In addition, only two functional SNPs in VEGFR-2 were examined in our study; these may not represent the complete genetic variability of the VEGFR-2 gene.

## 5. Conclusion

The present study showed that genetic variations in the KDR gene are associated with glioma formation in a Han Chinese population. In particular, rs2071559 and rs2239702 were associated with a higher risk of glioma, indicating that the KDR may act as a suppressor of tumor progression. However, other SNPs in KDR or other genes may also be important in the studied population. Genome-wide association studies of gliomas in a Chinese population are needed to discover all susceptibility loci and identify populations susceptible to gliomas. Early detection of glioma-susceptible individuals and risk factor monitoring are future research directions. In addition, more complex and systematic methods need to be established to analyze the etiological patterns of genes associated with a variety of environmental factors.

## Figures and Tables

**Table 1:** The demographical features at baseline in all patients.

Characteristics	Case group (*n* = 465)	Control group (*n* = 527)	*P*
Gender, *n* (%):			0.327
Men	272 (58.5)	292 (55.4)	
Women	193 (41.5)	235 (44.6)	
Age, years, means (SD)	42.22 (15.46)	40.20 (16.30)	0.046
Age group, *n* (%):			<0.001
<18 years	33 (7.1)	29 (5.5)	
18∼39 years	156 (33.5)	255 (48.4)	
40∼59 years	208 (44.7)	157 (29.8)	
≥18 years	68 (14.6)	86 (16.3)	
Cigarette smoking^a^, *n* (%):			0.572
Never	258 (55.5)	333 (63.2)	
Ever	59 (12.7)	63 (12.0)	
Current	109 (23.4)	127 (24.1)	
Missing data	39 (8.4)	4 (0.7)	
Family history of cancer, *n* (%):			0.378
No	344 (74.0)	424 (80.5)	
Yes	82 (17.6)	87 (16.5)	
Missing data	39 (8.4)	16 (3.0)	

**Table 2:** The clinical features at baseline in all patients.

	*n*	Percentage (%)
*Histologic-type, n (%)*:		
Astrocytic glioma	173	37.2
Glioblastoma	159	34.2
Oligodendroglioma	47	10.1
Ependymoma	67	14.4
Mixed glioma	19	4.1

*WHO classification, n (%)*:		
I	49	10.5
II	161	34.6
III	91	19.6
IV	164	35.3

**Table 3:** The observed allele frequencies by using SNP information.

	rs2071559	rs2239702
Chromosome	4	4
Location on chromosome	53,494,277	53,494,050
*P *_1_	0.4244	0.7115
Risk allele	C	A

*MAF*:		
Case group	0.386	0.189
Control group	0.302	0.1448
Database	0.300	0.116
*P*_2_	<0.001	0.014

MAF: minor allele frequency; Database: MAF for Chinese from HapMap databases; *P*_1_: *P* value for Hardy–Weinberg Equilibrium; *P*_2_: *P* value for difference in allele frequency distributions between cases and controls.

**Table 4:** The univariate analysis: results of gene frequency comparison between two groups.

	*Frequency*			*Unadjusted*	
	Case	Control	*P*	OR (95% CI)	*P*
*1559*					
Genotype, *n* (%)			0.001		
TT	180 (38.7)	261 (49.5)		1.00	
CT	211 (45.4)	214 (40.6)		1.43 (1.09, 1.87)	0.009
CC	74 (15.9)	52 (9.9)		2.06 (1.38, 3.09)	<0.001
Allele, *n* (%)			<0.001		
T	571 (61.4)	736 (69.8)		1.00	
C	359 (38.6)	318 (32.2)		1.46 (1.21, 1.75)	<0.001
Dominant, *n* (%)			0.001		
CC + CT	285 (61.3)	266 (50.5)		1.00	
TT	180 (38.7)	261 (49.5)		1.55 (1.21, 2.00)	0.001
Recessive, *n* (%)			0.005		
CT + TT	391 (84.1)	475 (90.1)		1.00	
CC	74 (15.9)	52 (9.9)		1.73 (1.18, 2.53)	0.004

*9702*					
Genotype, *n* (%)			0.024		
GG	315 (67.7)	384 (72.9)		1.00	
GA	124 (26.7)	130 (24.7)		1.16 (0.87, 1.55)	0.304
AA	26 (5.6)	13 (2.5)		2.44 (1.23, 4.82)	0.008
Allele, *n* (%)			0.014		
G	754 (81.1)	898 (85.2)		1.00	
A	176 (18.9)	156 (14.8)		1.34 (1.06, 1.70)	0.014
Dominant, *n* (%)			0.075		
GG	315 (67.7)	384 (72.9)		1.00	
GA + AA	150 (32.3)	143 (27.1)		1.28 (0.97, 1.68)	0.078
Recessive, *n* (%)			0.014		
GG + GA	439 (94.4)	514 (97.5)		1.00	
AA	26 (5.6)	13 (2.5)		2.34 (1.19, 4.61)	0.012

**Table 5:** The multivariate analysis.

	*Model 1*		*Model 2*	
	OR (95% CI)	*P*	OR (95% CI)	*P*
*KDR-rs2071559*:				
Genotype, *n* (%)				
TT	1.00		1.00	
CT	1.44 (1.10, 1.88)	0.008	1.45 (1.10, 1.92)	0.009
CC	2.09 (1.40, 3.13)	<0.001	2.04 (1.35, 3.09)	0.001
Dominant, *n* (%)				
TT	1.00		1.00	
CC + CT	1.56 (1.21, 2.02)	0.001	1.57 (1.20, 2.04)	0.001
Recessive, *n* (%)				
CT + TT	1.00		1.00	
CC	1.75 (1.19, 2.56)	0.004	1.69 (1.15, 2.50)	0.008

*KDR-rs2239702*:				
Genotype, *n* (%)				
GG	1.00		1.00	
GA	1.16 (0.87, 1.55)	0.303	1.12 (0.83, 1.51)	0.447
AA	2.54 (1.28, 5.05)	0.008	2.50 (1.25, 5.01)	0.010
Dominant, *n* (%)				
GG	1.00		1.00	
GA + AA	1.29 (0.98, 1.69)	0.072	1.26 (0.95, 1.66)	0.116
Recessive, *n* (%)				
GG + GA	1.00		1.00	
AA	2.44 (1.24, 4.83)	0.010	2.63 (1.34, 5.35)	0.011

## Data Availability

The data supporting the findings of this study are available within the article.
